# Supplementary material for: Identifying the origin of delayed electroluminescence in a polariton organic light-emitting diode
Source: Nanophotonics. 2024 Jan 8;13(14):2565–73. doi: 10.1515/nanoph-2023-0587 (PMC11147497; doi:10.1515/nanoph-2023-0587)
Supplement: Supplementary file 1 — Supplementary Material Details [file j_nanoph-2023-0587_suppl_001.pdf]

Supporting Information

Identifying the origin of delayed electroluminescence in a polariton organic light-emitting diode

Ahmed Gaber Abdelmagid, Hassan A. Qureshi, Michael A. Papachatzakis, Olli Siltanen, Manish Kumar, Ajith Ashokan, Seyhan Salman, Kimmo Luoma, and Konstantinos S. Daskalakis
Corresponding authors: olmisi@utu.fi (O. Siltanen), konstantinos.daskalakis@utu.fi (K. S. Daskalakis)

Contents

Supplementary Figure S1. Density functional theory simulations.

Supplementary Figure S2. Angle-resolved reflectivity of the reference device and POLEDs 1–3.

Supplementary Figure S3. Comparison between top- and bottom-emitting POLED.

Supplementary Figure S4. Steady-state and time-resolved measurements of the reference device.

Supplementary Figure S5. Comparison of EL intensity between TDAF photoluminescence and POLEDs.

Supplementary Figure S6. Measurement setup.

Supplementary Figure S7. Degradation effect in time-resolved electroluminescence.

Supplementary Figure S8. Current-dependent time-resolved electroluminescence of POLED 1 and 2.

Supplementary Figure S9. Fitting residuals of the different mechanisms on POLED 2.

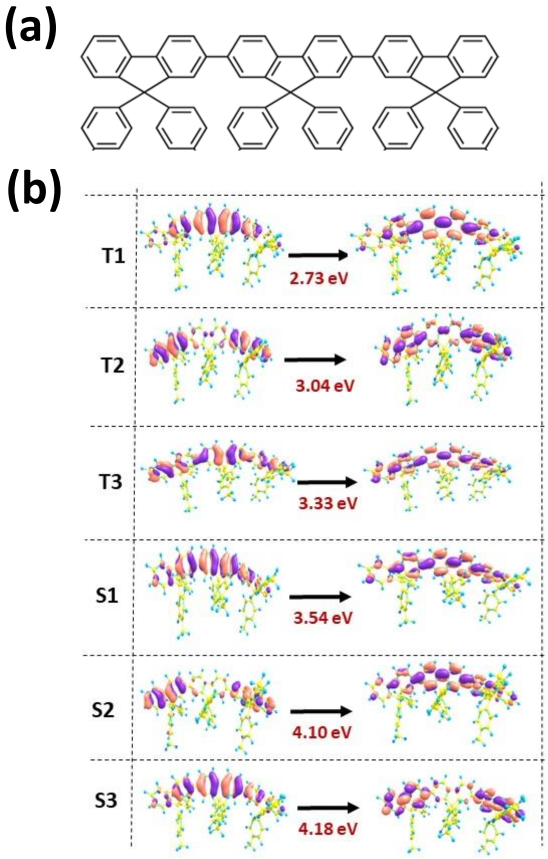

(c)

SOC Actual (cm^{-1})	
	SRSH (ε=3.5)
S1-T1	0.019
S1-T2	0.104

(d)

	E (abs)	E (emit)	E (0-0)
S_1	3.54	2.99	3.22
T_1	2.73	2.05	2.38
ΔE_{ST}	0.81	0.94	0.84

Fig. S1: (a) The molecular structure of TDAF. (b) Natural transition orbitals (NTOs) offer a representation of the wavefunctions of the hole and electron within a specific excited state, in the first three excited levels of the singlet and triplet states. The overlap between the hole and electron NTOs results in S_1 exhibiting characteristics of local excitation (LE), which implies the strong transition of the TDAF exciton. (c) Calculated spin-orbit coupling matrix elements. (d) Calculated vertical (abs), adiabatic (emit), and potential surface minimum (0-0) energies for the first singlet and triplet excited states in eV. These calculations were performed at Screened Range Separated Hybrid functional LC-whPBE with an implicit dielectric constant of 3.5. We opt for this approach as it offers the best alignment between our calculations, the earlier experimental findings of TDAF [22], and our results.

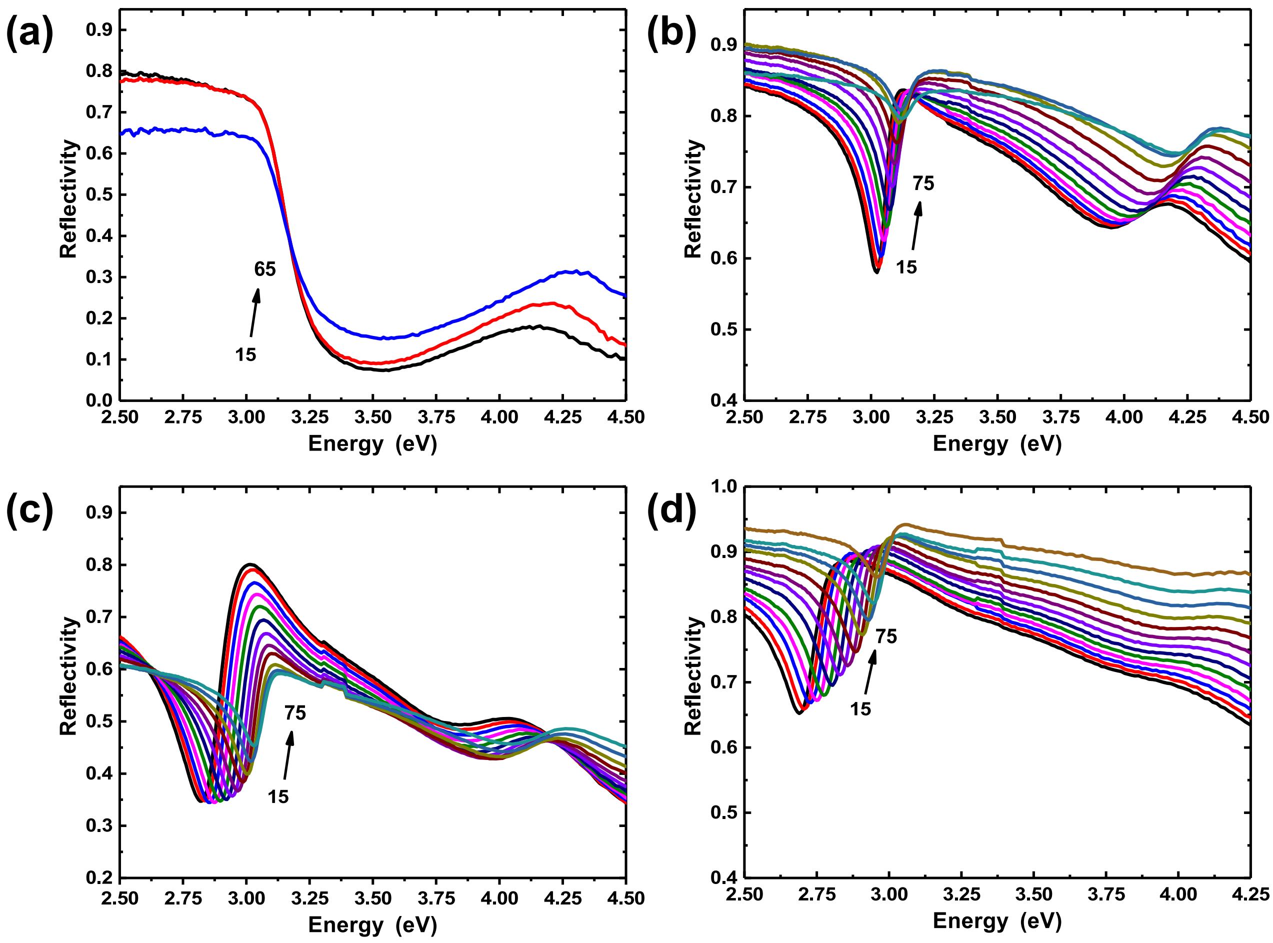

Fig. S2: Angle-resolved reflectivity for (a) ITO-based device with the angle interval 25°—the data shows an angle independent spectrum which is similar to the emission of the TDAF molecule, i.e., no strong coupling effect—(b) POLED 1 with the angle interval 5°, (c) POLED 2 with the angle interval 5°, and (d) POLED 3 with the angle interval 5°.

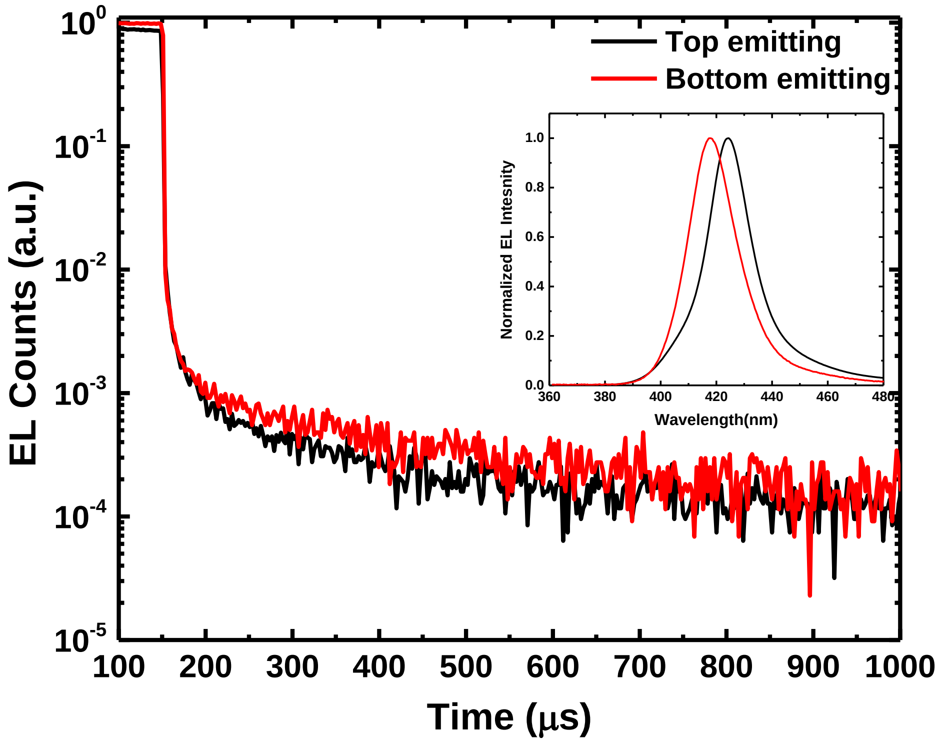

Fig. S3: Normalized time-resolved EL counts for a top-emitting POLED (black) consisting of an aluminium bottom contact (70 nm), MoO_3 hole injection layer (5 nm), TDAF emitting layer, LiF electron injection layer (1 nm), and an aluminium top contact (25 nm) and a bottom-emitting POLED (red) consisting of an aluminium bottom contact (30 nm), MoO_3 hole injection layer (5 nm), TDAF emitting layer, LiF electron injection layer (1 nm), and an aluminium top contact (100 nm). The inset shows the normalized EL spectra of both POLEDs. The slight difference in the delayed part of the EL is due to the difference in the top and bottom contact thicknesses of the two POLEDs.

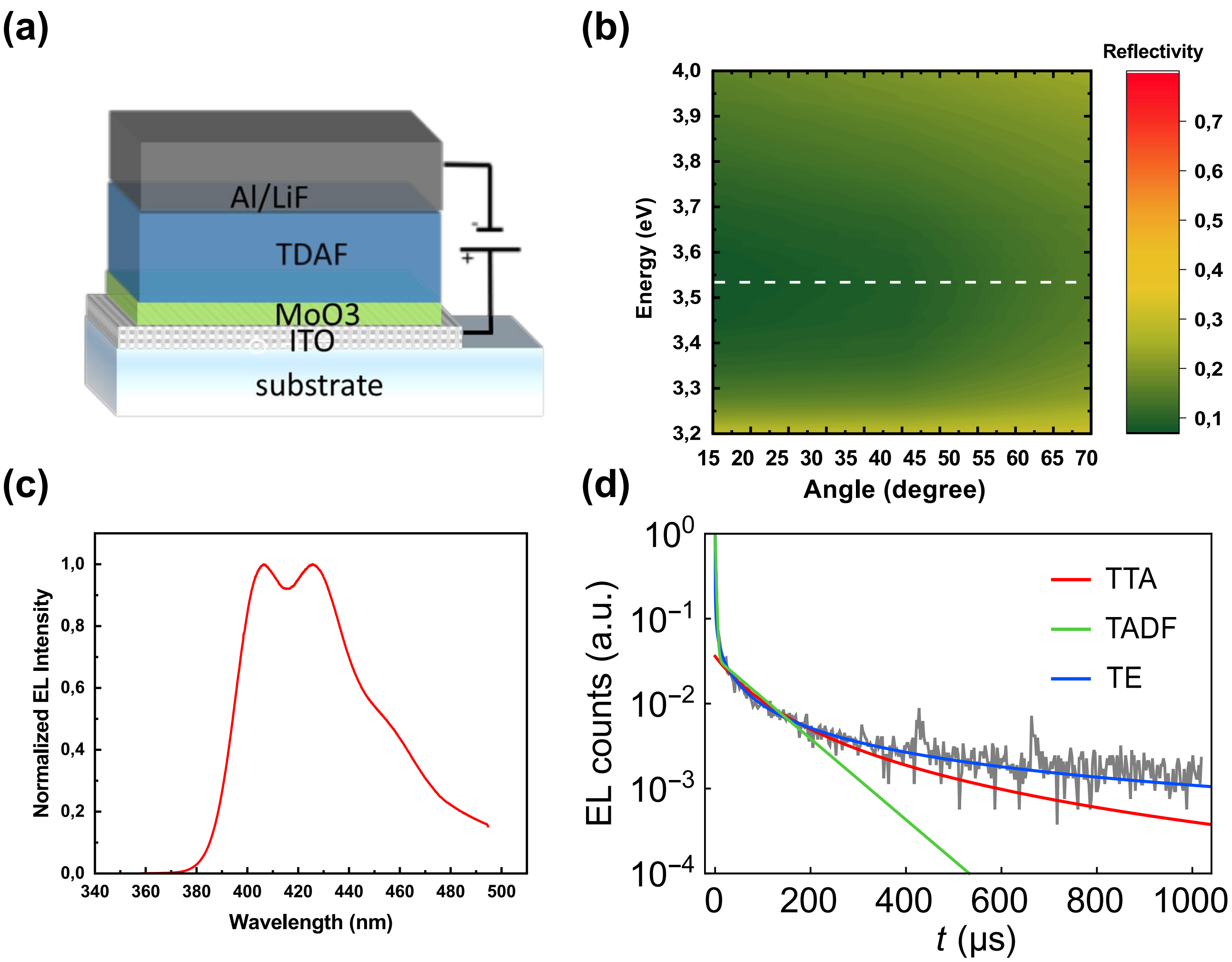

Fig. S4: (a) Schematic representation of the reference device. (b) Angle-resolved reflectivity of the reference device. (c) Normalized EL spectrum of the reference device. (d) Normalized time-resolved EL counts of the reference device at $J = 75$ mA/cm^2 (grey) and the fitted models. Here, the RISC related to TADF happens from T_1 to S_1 (not LP). As expected, the model fits poorly.

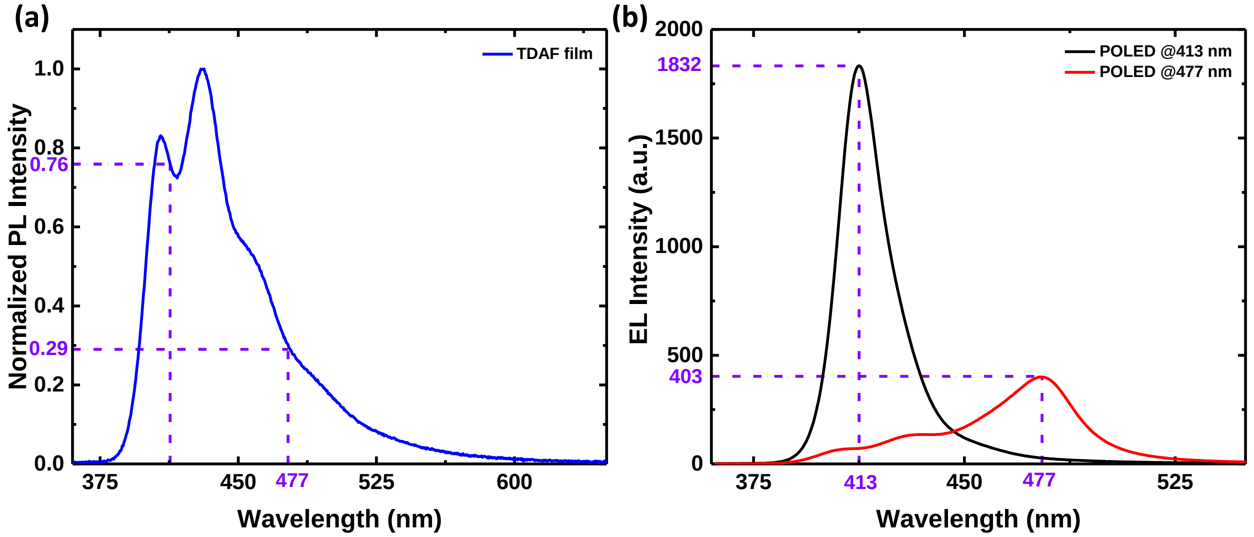

Fig. S5: (a) Normalized PL spectrum of the bare TDAF film. (b) EL spectra of two different detunings of POLEDs. The mentioned wavelengths are the wavelengths of the peaks of the polaritons.

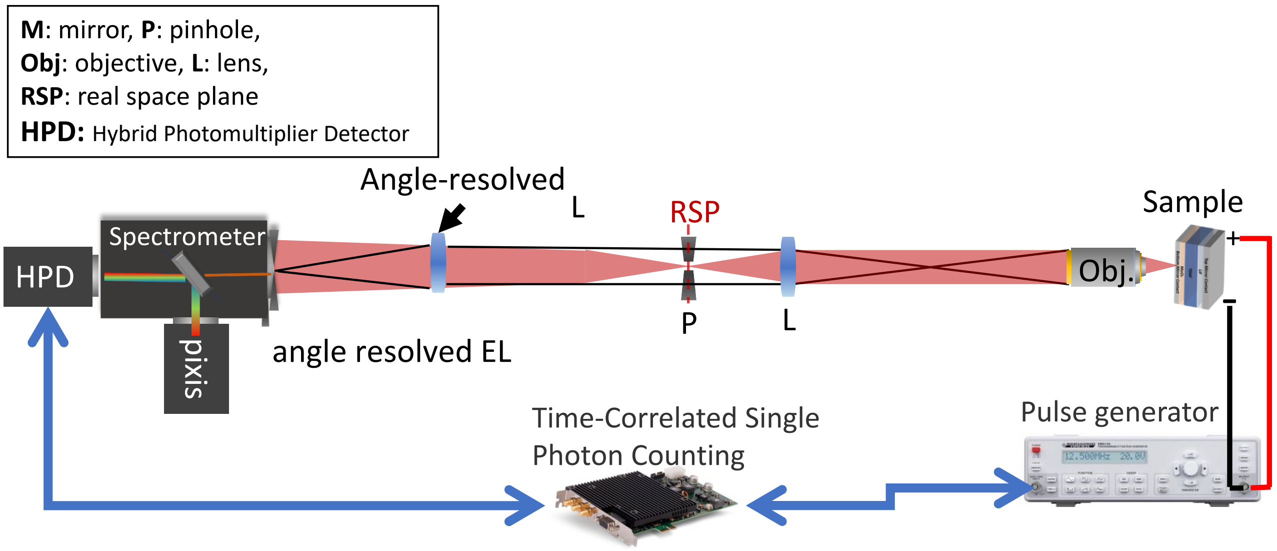

Fig. S6: Schematic representation of the measurement setup. The electroluminescence (EL) was collected with a Nikon CFI Plan Apochromat Lambda 4X magnification and 0.2 NA. A 200 mm tube lens was used to focus the EL image of the samples on an iris to spatially filter out emission from the sides of the OLED. The filtered image was transferred by a 200 mm relay lens to the slit of the spectrometer (Acton 300 Princeton Instruments) which has two outputs that can be switched via a mirror in the spectrometer. To verify the EL spectrum, the EL was directed to a 2D CCD (PIXIS:400B Princeton Instrument). To perform the TCSPC measurement, EL from the LP band-bottom was spectrally filtered 6 nm around the LP peak and was directed to the hybrid photomultiplier detector (PMA Hybrid PicoQuant) output. Current injection to the samples was realized by a pulse generator (HM8150 Rohde & Schwarz) with 250 square pulses with 9 ns turn-on time. We used a TimeHarp 260 TCSPC board module (PicoQuant) to track the photons and synchronize the pulse generator.

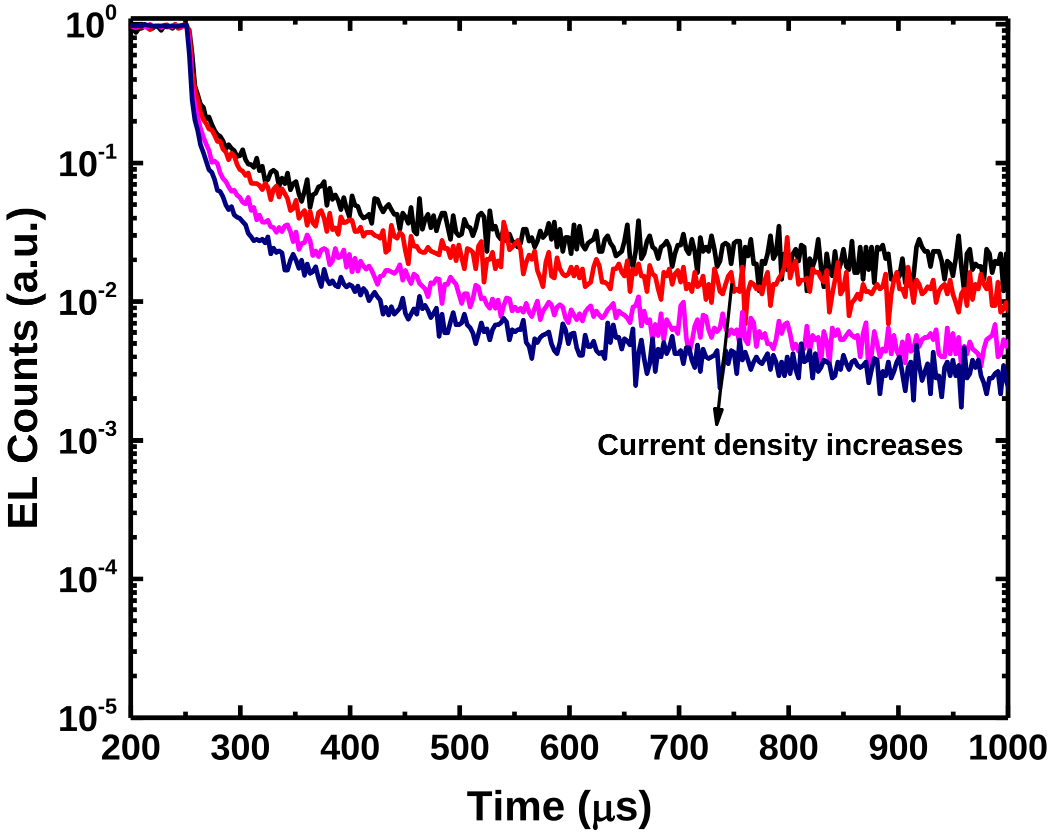

Fig. S7: Normalized time-resolved EL counts for a degraded POLED. The TE has increased as a result of the sample's deterioration subsequent to its exposure to ambient air over a period of time.

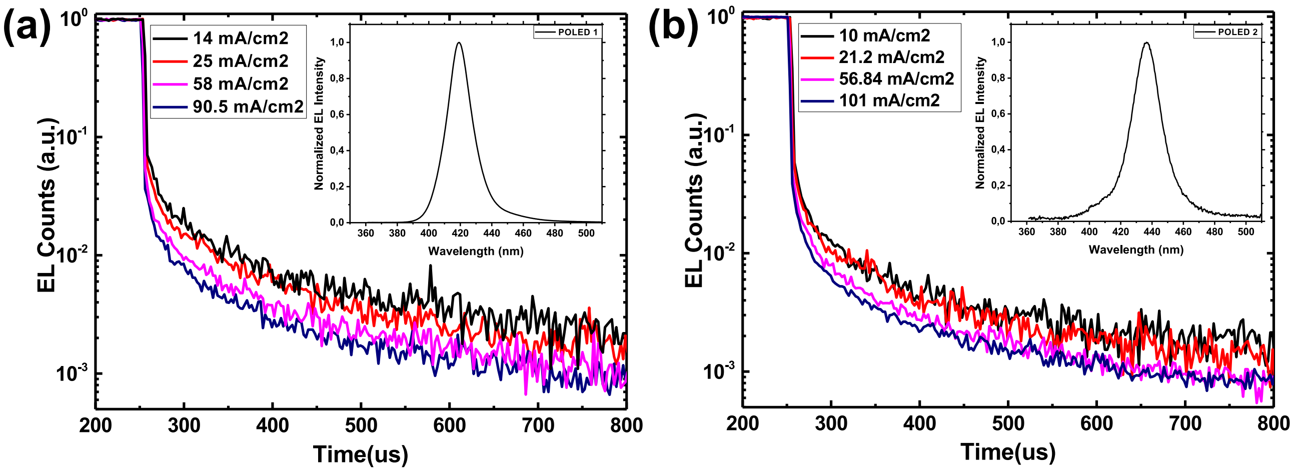

Fig. S8: Normalized time-resolved EL from (a) POLED 1 and (b) POLED 2. The insets are the normalized EL spectra.

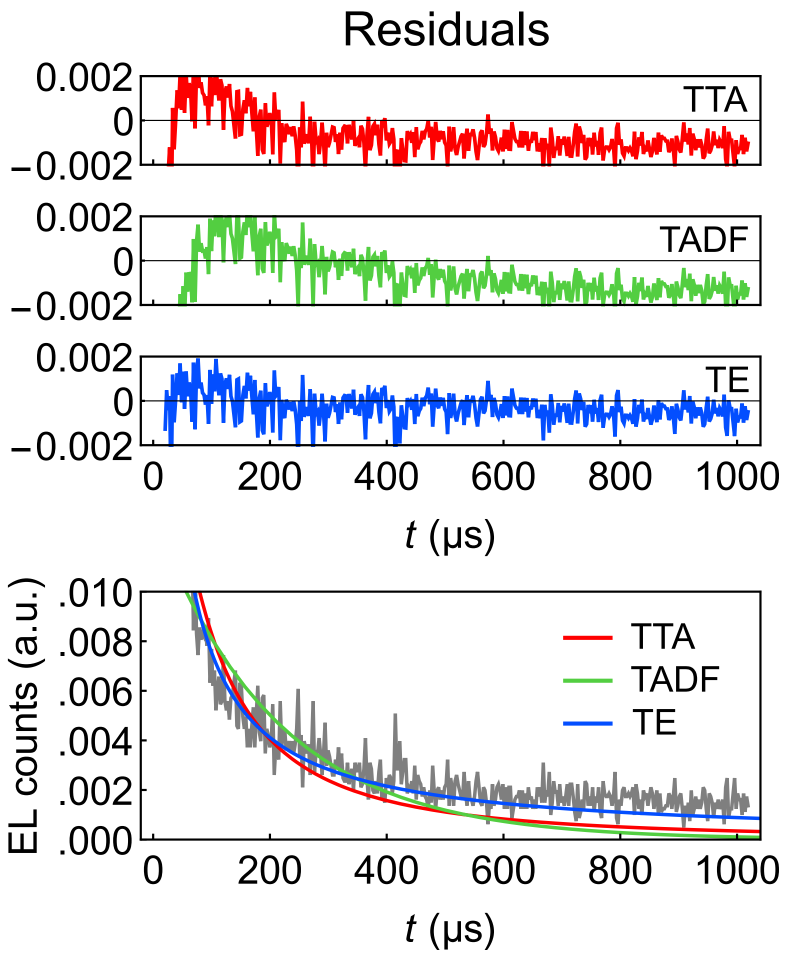

Fig. S9: Residuals of the different models of delayed EL with POLED 2 and $J = 6.31$ mA/cm^2.
